# Bacterial Cell Wall Quality Control during Environmental Stress

**DOI:** 10.1128/mBio.02456-20

**Published:** 2020-10-13

**Authors:** Elizabeth A. Mueller, Petra Anne Levin

**Affiliations:** a Department of Biology, Washington University in St. Louis, St. Louis, Missouri, USA; b Center for Science & Engineering of Living Systems (CSELS), McKelvey School of Engineering, Washington University in St. Louis, St. Louis, Missouri, USA; University of Texas Health Science Center at Houston

**Keywords:** adaptation, antibiotics, peptidoglycan, cell wall, stress response

## Abstract

Single-celled organisms must adapt their physiology to persist and propagate across a wide range of environmental conditions. The growth and division of bacterial cells depend on continuous synthesis of an essential extracellular barrier: the peptidoglycan cell wall, a polysaccharide matrix that counteracts turgor pressure and confers cell shape. Unlike many other essential processes and structures within the bacterial cell, the peptidoglycan cell wall and its synthesis machinery reside at the cell surface and are thus uniquely vulnerable to the physicochemical environment and exogenous threats. In addition to the diversity of stressors endangering cell wall integrity, defects in peptidoglycan metabolism require rapid repair in order to prevent osmotic lysis, which can occur within minutes. Here, we review recent work that illuminates mechanisms that ensure robust peptidoglycan metabolism in response to persistent and acute environmental stress. Advances in our understanding of bacterial cell wall quality control promise to inform the development and use of antimicrobial agents that target the synthesis and remodeling of this essential macromolecule.

## Abstract (precis)

Nearly all bacteria are encased in a peptidoglycan cell wall, an essential polysaccharide structure that protects the cell from osmotic rupture and reinforces cell shape. The integrity of this protective barrier must be maintained across the diversity of environmental conditions wherein bacteria replicate. However, at the cell surface, the cell wall and its synthesis machinery face unique challenges that threaten their integrity. Directly exposed to the extracellular environment, the peptidoglycan synthesis machinery encounters dynamic and extreme physicochemical conditions, which may impair enzymatic activity and critical protein-protein interactions. Biotic and abiotic stressors—including host defenses, cell wall active antibiotics, and predatory bacteria and phage—also jeopardize peptidoglycan integrity by introducing lesions, which must be rapidly repaired to prevent cell lysis. Here, we review recently discovered mechanisms that promote robust peptidoglycan synthesis during environmental and acute stress and highlight the opportunities and challenges for the development of cell wall active therapeutics.

## INTRODUCTION

The growth and survival of single-celled organisms hinge on their ability to adapt to dynamic, and potentially hostile, environmental conditions. Effective adaptation requires a means to maintain essential cellular processes in the face of diverse endogenous and exogenous stress. For bacteria, preserving the integrity of an essential extracellular barrier—the peptidoglycan (PG) cell wall—is often the difference between life and death.

**FIG 1:**
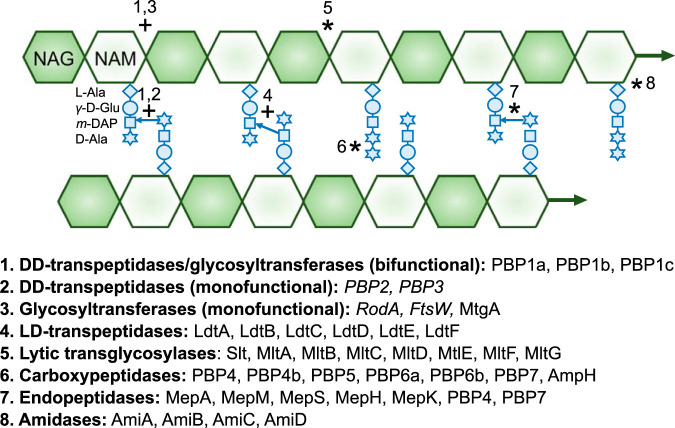
Summary of E. coli extracellular peptidoglycan enzymes and their activities. Schematic of the PG cell wall depicting major synthesis reactions (numbered 1 to 4 and indicated by a plus symbol) and autolysis reactions (numbered 5 to 8 and indicated by an asterisk) and associated enzymes. Essential enzymes are shown in italic type. m-DAP, *meso*-diaminopimelic acid.

A continuous macromolecule encasing the plasma membrane, PG consists of glycan strands of repeating β(1,4)-linked *N*-acetylglucosamine (NAG) and *N*-acetylmuramic acid (NAM) disaccharides and short peptide stems, which are covalently attached to the NAM sugars. Peptide stems from adjacent glycan strands are cross-linked to one another to create a PG matrix collectively referred to as the sacculus (Fig. 1). When intact, the PG sacculus protects cells from osmotic rupture and reinforces cell shape and size (1, 2). Yet the line between safeguard and liability is thin: sustained breaches in the sacculus are catastrophic and lead to rapid cell lysis. The vulnerability of PG and its synthesis machinery is exploited by antibacterial host defenses (e.g., lysozyme) and cell wall active antibiotics (e.g., β-lactams and glycopeptides). Although more mundane, suboptimal growth conditions (e.g., pH stress or metal limitation) also threaten PG integrity by interfering with the activity of PG synthesis enzymes and the interactions between them.

Here, we briefly review PG biology, then detail the mechanisms which preserve PG integrity in response to environmental and acute stress. We focus on strategies in Escherichia coli because it is the predominant model organism for studying cell wall biogenesis and is an environmental generalist, but we also draw examples from other bacteria to highlight conservation and diversity when relevant. A clear understanding of the mechanisms that ensure robust PG homeostasis across environmental conditions is essential to developing new cell wall active drugs and preserving the value of our current arsenal.

## PEPTIDOGLYCAN METABOLISM

### Peptidoglycan precursor biogenesis.

PG synthesis begins in the cytoplasm. Soluble nucleotide-activated sugars (UDP-*N-*acetylglucosamine and UDP-*N*-acetylmuramyl pentapeptide) are assembled on a 55-carbon carrier lipid (undecaprenyl phosphate) to form a lipid-linked disaccharide precursor (lipid II) (3). Flippases, including MurJ and Amj, translocate lipid II to the outer leaflet of the plasma membrane (4, 5). In the periplasm of Gram-negative bacteria or at the cell surface of Gram-positive bacteria, the disaccharide subunits are polymerized, liberated from undecaprenyl phosphate, and assembled into the existing PG matrix through the coordinated actions of cell wall synthases and autolysins (6).

### Peptidoglycan synthases.

PG synthases are categorized by their ability to perform glycosyl transfer (glycan polymerizing) and/or transpeptidation (peptide cross-linking) reactions. Two classes of transpeptidases form distinct linkages between the adjacent peptide stems: dd-transpeptidases catalyze cross-links between the third and fourth amino acid positions (3-4 cross-links), whereas ld-transpeptidases catalyze cross-links between the third positions of the two stems (3-3 cross-links) (Fig. 1). Although inactivated by carbapenems (7, 8), ld-transpeptidases are insensitive to penicillin and closely related β-lactam antibiotics (8). In contrast, penicillin binds to and inhibits the activity of dd-transpeptidases (9). dd-Transpeptidases are therefore referred to as penicillin binding proteins (PBPs). Cells encode two major classes of PBP synthases: class A and class B PBPs.

Class A PBPs are bifunctional enzymes, possessing both glycosyltransferase and dd-transpeptidase activities. E. coli genes encode three class A PBPs—PBP1a, PBP1b, and PBP1c. PBP1a and PBP1b share an essential, but unclear, role in PG biogenesis (10) and are believed to function outside cytoskeleton-directed cell wall synthesis machines (discussed below) (11). The activity of PBP1a and PBP1b is mediated through interaction with a cognate outer membrane lipoprotein cofactors LpoA and LpoB, respectively (12, 13). PBP1c, in contrast, has no known role in PG synthesis under standard laboratory conditions and cannot compensate for the combined loss of PBP1a and PBP1b (14). Intriguingly, PBP1c is encoded downstream of a α_2_-macroglobulin involved in protection against host proteases. It is tempting to speculate that PBP1c may have a role in PG synthesis in host-relevant niches (15, 16).

Class B PBPs are monofunctional dd-transpeptidases, whose activity is specifically coupled to a monofunctional glycosyltransferase belonging to the SEDS (shape, elongation, division, and sporulation) family (17–19). E. coli genes encode two essential class B PBP/SEDS enzyme pairs—PBP2/RodA and PBP3/FtsW—that function as parts of multicomponent PG synthesis complexes involved in cell elongation and cell division, respectively. PBP2 and RodA associate with membrane proteins RodZ, MreC, and MreD and small patches of the actin homolog MreB to form the Rod system (“elongasome”). The Rod system elongates the cell by inserting new PG material in the lateral cell body throughout the cell cycle and is required for rod shape (20–24). Analogously, PBP3 (FtsI) and FtsW are essential components of the cytokinetic ring (“divisome”), a multicomponent PG synthesis complex scaffolded by treadmilling filaments of the tubulin homolog FtsZ (25, 26). Unlike PBP2/RodA, PBP3/FtsW activity is spatially confined to midcell and temporally restricted to cytokinesis (19, 26, 27). Genetic or chemical inactivation of PBP3 and/or FtsW inhibits cell division and leads to the formation of long filamentous cells (28–31).

In addition to the PBP and SEDS synthases, E. coli genes encode seven nonessential monofunctional enzymes involved in PG synthesis, including six penicillin-insensitive ld-transpeptidases and one glycosyltransferase. ld-transpeptidases LdtA, LdtB, and LdtC anchor Braun’s lipoprotein to the PG (32), whereas LdtD, LdtE, and LdtF promote the formation of 3-3 cross-links between peptide stems (32, 33), particularly during stationary-phase growth (34). Finally, E. coli carries a gene that encodes a single monofunctional glycosyltransferase, MtgA, with an unknown role in PG biogenesis (35).

### Peptidoglycan autolysins.

PG hydrolases and lyases, collectively referred to as “autolysins,” cleave nearly every bond within the PG sacculus. Major autolysins and their cleavage sites include dd-endopeptidases (peptide cross-link), lytic transglycosylases (glycosidic bond), dl-endopeptidases and carboxypeptidases (peptide bond within stem peptide), and amidases (amino-sugar bond) (Fig. 1). The functions of autolysins are as varied as their biochemical activities and even differ among enzymes within the same class or between homologs in different bacteria (36). Functional assignment is also impeded by significant apparent redundancy among the autolysins, with up to eight enzymes capable of catalyzing each reaction (discussed below). In E. coli, PG autolysins are implicated in growth, cell separation, morphogenesis, and PG maturation.

Linkages between glycan strands must be broken in order to expand and divide the sacculus during normal growth. In E. coli, three semiredundant dd-endopeptidases—MepA, MepS, and MepM—hydrolyze cross-links between existing glycan strands to make space for insertion of nascent PG into the sacculus (37, 38). Cell separation during cytokinesis also requires the septal PG to be split to form the new poles of the daughter cells. Cell separation in E. coli is primarily achieved through the combined activity of three LytC-type *N*-acetylmuramoyl-l-alanine amidases (AmiA, AmiB, and AmiC), which cleave the peptide stem from the glycan backbone of PG to produce stemless (“denuded”) glycans (39, 40). Certain endopeptidases and lytic transglycosylases may also assist in cell separation (41–43).

Autolysins also dictate PG processing and maturation. Carboxypeptidases trim the pentapeptide to four or three amino acids in length, thereby dictating the types of cross-links that can be formed at a particular stem. Like the PBP synthases, many carboxypeptidases also bind to penicillin and are named accordingly. Carboxypeptidase activity influences cell morphogenesis: E. coli cells impaired for the production of the major carboxypeptidase PBP5 bulge, kink, bend, and branch (44, 45). Lytic transglycosylases, on the other hand, cleave the β(1,4) glycosidic bond between disaccharides in a nonhydrolytic reaction. These enzymes influence glycan chain length (46) and contribute to PG turnover, which can account for up to 50% of the total PG material per generation in E. coli (47, 48).

## RESPONSES TO ENVIRONMENTAL THREATS

Many bacteria persist and propagate across a wide range of chemical, physical, and nutritional environments and must consequently maintain cell wall integrity across diverse conditions. Enteric bacteria like E. coli, for example, infiltrate their mammalian hosts through contaminated food products (ambient temperature, variable pH) and then pass through the oral cavity and upper gastrointestinal tract (body temperature, acidic pH) before colonizing the colon (neutral to basic pH). Upon exiting the gut, certain E. coli isolates infect and persist in the upper and lower urinary tract (49)—an osmotic-, nutrient-, and pH-variable environment dependent on the host’s diet and hydration state.

While most essential processes (e.g., DNA replication, transcription, and translation) are confined to the cytosol, where homeostatic control systems limit environmental fluctuations by buffering and maintaining the salt content in response to pH and osmotic stress (50, 51), many essential reactions involved in PG biogenesis take place exterior to the plasma membrane. These reactions at the cell surface are thus uniquely exposed to the extracellular environment (2). Although PG precursors are generated within the stable confines of the cytosol, the subunits are assimilated into the existing PG macromolecule through reactions that take place in the extracellular space of Gram-positive bacteria and periplasm of Gram-negative bacteria (Fig. 1). Both of these compartments assume many of the physical and chemical properties of the environment (e.g., pH and osmolarity) due to either the complete absence or porosity of a secondary lipid bilayer (52–54).

Environmental fluctuations may challenge the biochemistry of PG biogenesis by disrupting enzymatic activity and/or regulatory protein-protein interactions, both of which are generally optimized for a particular set of reaction conditions. Nevertheless, laboratory E. coli proliferates in environments which range from pH 4 to 9 and 0 to 0.8 M NaCl with only modest differences in overall PG composition (55–57), indicating the existence of mechanisms to promote robust cell wall biogenesis. As discussed below, these mechanisms extend beyond canonical stress response pathways and signal transduction networks and instead involve shifts in enzymatic activity and PG complex composition, allowing for near-instantaneous responses to environmental stimuli.

Below we elaborate the different strategies bacteria employ to ensure cell wall metabolism keeps pace with cell growth across environmental conditions. We focus on two of the adaptations that promote PG homeostasis during steady-state growth in nonstandard conditions (i.e., constant stress): (i) plasticity within the active repertoire of cell wall enzymes, and (ii) production of modulatory factors that stabilize multicomponent cell wall synthesis complexes. Mechanisms that protect the cell wall in response to acute (non-steady-state) treats will be addressed in the subsequent section.

### Apparent redundancy.

Sets of PG enzymes with overlapping activity profiles appear to be a major mechanism to preserve cell wall integrity across environmental conditions. Redundancy is specific among the extracellular repertoire of enzymes, which are directly exposed to the extracellular environment. Whereas PG precursors are synthesized with a nearly 1:1 stoichiometry between reactions and enzymes (10 reactions, 12 enzymes), there are over 37 enzymes that carry out the eight reactions involved in the E. coli PG metabolism in the periplasm (Fig. 1). Of these extracellular enzymes, only four are essential. The class B PBP/SEDS protein pairs, PBP2-RodA and PBP3-FtsW, are required for cell elongation and division, respectively (6). Individual loss of any of the remaining 33 enzymes fails to impact E. coli growth or morphology under standard culture conditions. In fact, some mutants defective for multiple enzymes of the same class still do not exhibit phenotypes in the laboratory setting (37, 39, 41, 45, 58). E. coli is not unique in its redundant extracellular cell wall enzymes. Bacillus subtilis genes encode over 45 extracellular PG synthases and autolysins. Only three enzymes—including RodA, FtsW, and PBP2b—are essential for vegetative growth under standard culture conditions (6).

Apparent redundancy in the extracellular reactions of PG biogenesis scales with environmental complexity. Obligate mammalian and obligate intracellular pathogens carry genes that encode fewer extracellular PG enzymes than environmental generalists that inhabit a range of ecological niches (e.g., E. coli) or organisms that undergo complex development lifecycles (e.g., spore-forming B. subtilis). Phylogenetic analysis of PG synthesis enzymes indicates the presence of 16 and 21 putative transpeptidases and glycosyltransferases in the genomes of E. coli and B. subtilis, respectively (59). The obligate intracellular pathogen Chlamydia trachomatis and human gut specialist Helicobacter pylori, in contrast, carry genes that encode as few as three enzymes each. Obligate mammalian pathogens that are capable of infecting diverse host niches (e.g., Staphylococcus aureus and Streptococcus pyogenes) carry genes that encode an intermediate number of PG synthesis enzymes (59).

Altogether, the relationship between apparent redundancy and the environment suggests that some enzymes may be “specialized” and preferentially used during growth under particular environmental conditions. Rather than a single set of machinery synthesizing and remodeling PG across all growth conditions, the repertoire of active cell wall synthesis enzymes may be dynamic with respect to the environment, and their cumulative activity allows for viability across the full range of growth-permissive environments (60) (Fig. 1). Consistent with this model, a multitude of enzymes with condition-dependent changes in production, activity, and essentiality has been identified in the last 5 years (Table 1). It is important to point out, however, that environmental specialization may not explain all redundancies in PG enzymes; in some cases, apparent redundancy can be attributed to differences in biochemical activity, subcellular localization, cell cycle regulation, and/or interaction partners (examples reviewed in references 36 and 61).

**TABLE 1:** Peptidoglycan genes and proteins with condition-dependent phenotypes

Organism	Function	Activity	Protein	Remarks
*Escherichia coli*	Precursor synthesis	Enterobacterial common antigen recycling	ElyC	Required for growth at low temp (30°C) (118)
	Cell wall synthesis	Class A PBP	PBP1a	Required for maximal growth rate in alkaline pH (pH 6.9 to 8.2) (57)
				Impaired activity in acidic medium (pH 4.8) (57)
				Aberrant localization pattern in acidic medium (pH 5.2) (57)
		Class A PBP	PBP1b	Required for maximal growth rate in acidic pH (pH < 5.5) (57)
				Upregulated during outer membrane stress (33, 117)
				Required for survival during mechanical stress (95, 98)
		ld-Transpeptidase	LdtD	Upregulated during outer membrane stress (33, 117)
	Cell wall hydrolysis	dd-Carboxypeptidase	PBP6a	Upregulated during outer membrane stress (33, 117)
		dd-Carboxypeptidase	PBP6b	Upregulated in acidic medium (pH 5.0) (55)
				Increased stability in acidic medium (pH 5.0) (55)
				Increased sp act in acidic medium (pH 5.0) (55)
		dd-Endopeptidase	MepS	Required for maximal growth rate in acidic pH (pH < 5.5) (57)
		Lytic transglycosylase	MltA	Required for maximal growth rate in acidic pH (pH < 5.5) (57)
				Increased activity *in vitro* at acidic pH (pH 4.0 to 4.5) (119)
				Increased activity *in vitro* and *in vivo* at low temp (30°C) (119, 120)
			MltG	Required for maximal growth rate in alkaline pH (pH 6.9 to 8.4) (57)
	Cell division	Regulation	FtsEX	Required for growth in low-osmotic-strength media (82)
		Regulation	FtsP	Required for growth in low-osmotic-strength media at high temp (42°C) (75)
				Required for growth during oxidative stress and DNA damage at high temp (42°C) (75)
		Regulation	FtsN	Hyperenriched at midcell during growth in acidic medium (pH < 5.5) (80)
*Vibrio cholerae*	Cell wall synthesis	Class A PBP	PBP1a	Required for maximal growth in stationary phase and minimal medium (121)
				Required for fitness in infection of the infant mouse small intestine (121)
	Cell wall hydrolysis	dd-Endopeptidase	ShyB	Zur-mediated upregulated in Zn-deplete medium (70)
				Activity resistant to Zn chelators *in vitro* (70)
		Regulation	NlpD	Specifically required for intestinal colonization (122)
				Required for resistance to bile salts (122)
*Acinetobacter baumannii*	Cell wall hydrolysis	dd-Carboxypeptidase and endopeptidase	ZrlA	Upregulated in Zn-deplete medium (71)
				Required for efficient colonization and dissemination in murine pneumonia model (71)
				Required for Zn uptake (71)
*Caulobacter crescentus*	Cell wall synthesis	dd-Transpeptidase	PBP2	Enriched at the midcell in low-osmotic-strength media (<40 mosmol/kg) (123)
*Pseudomonas aeruginosa*	Cell division	dd-Transpeptidase	PBP3x	Upregulated in stationary phase (68)
*Salmonella enterica*	Cell wall hydrolysis	dl-Endopeptidase	EcgA	Upregulated in epithelial cells and in acidic minimal medium (62)
				Required for fitness in murine typhoid model (62)
		Amidase	AmiA	Required for fitness in the murine inflamed gut (124)
			AmiC	Required for fitness in the murine inflamed gut (124)
	Cell division	Division-specific class B PBP	PBP3Sal	Upregulated in acidic medium (pH < 5.8), macrophages, and in murine infection model (63)
				Sufficient for cell division in acidic medium and in macrophages (63)
	Cell elongation	Elongation-specific class B PBP	PBP2Sal	Upregulated in acidic medium (pH < 5.8), macrophages, and in murine infection model (67)
*Mycobacterium tuberculosis*	Cell wall synthesis	Class A PBP	PonA2	Required for growth and rod morphology in stationary phase (125)
				Required for growth in anaerobic environments (125)
				Required for growth in acidic medium (66)
	Cell wall hydrolysis	Amidase	Ami1	Required for persistence in murine model (64)
		Endopeptidase	RipA	Required for cell growth in acidic medium (65)
				Required for persistence in murine model (64, 65)
				Regulated through acid-responsive protease MarP (65)
	Cell division	Regulation	FipA	PnkA-dependent phosphorylation is required for FtsZ localization to midcell during oxidative stress (126)
				Required for growth and division in macrophages (126)
	Cell division	Regulation	PerM	Required for persistence and cell division in murine model (88)
				Required for survival during acidic pH stress and Fe limitation (88)
*Bacillus subtilis*	Cell elongation	Cytoskeleton	YvcK (GlmR)	Compensates for MreB during growth on gluconeogenic carbon sources (127)
	Cell division	Class B PBP	PBP3	Can compensate for division in absence of PBP2B activity (128)
*Listeria monocytogenes*	Cell elongation	Glycosyltransferase	RodA3	Upregulated in response to cell envelope stress via CesRK (69)
	Cell division	Glycosyltransferase	FtsW2	Upregulated in response to cell envelope stress via CesRK (69)
*Staphylococcus aureus*	Cell wall synthesis	Regulation	MreC, MreD	Required for fitness in murine chronic abscess model (92)

**(i) Environmental pH influences activity of E. coli PG synthases and autolysins.** E. coli is exposed to a range of pH environments as it progresses through the gastrointestinal tract and when it persists in the urinary bladder. Accordingly, enzymes with pH-dependent changes in activity have been identified in nearly every class of “nonessential” E. coli cell wall synthases and autolysins.

Our recent work identified five enzymes required for E. coli fitness when cultured specifically in acidic or alkaline growth medium. We compared how pH influences the growth rate of 32 E. coli mutants, each defective for a single “nonessential” cell wall enzyme. The subset of pH-sensitive proteins identified spanned three enzymatic classes and included PBP1a and PBP1b, two class A PBPs that share an essential—but unclear—role in cell wall biogenesis under standard culture conditions. Loss of PBP1b attenuated growth or led to lysis at pH < 5.5. In contrast, cells defective for PBP1a exhibited a modest but significant growth defect between pH 6.9 and 8.2. The conditional fitness requirement of PBP1a and PBP1b is mediated in part through pH-dependent changes in activity: while PBP1b remains active across a range of pH conditions, PBP1a is inactive at pH 4.8 *in vitro* (57). Differential utilization of PBP1a and PBP1b appears to impact intrinsic resistance to certain β-lactam antibiotics. In acidic medium, E. coli is up to 64-fold more resistant to β-lactams that target PBP2 and PBP3 through a mechanism dependent on PBP1b activity (57).

Growth analysis, however, is not sufficient to identify the full repertoire of pH-sensitive cell wall enzymes. The Vollmer and Young groups identified the first identified pH specialist—the dd-carboxypeptidase PBP6b—by comparing muropeptide profiles and morphology of mutants defective for up to eight PG enzymes (55). This analysis revealed that PBP6b is capable of trimming pentapeptide stems and maintaining rod-shaped morphology in acidic (pH 5.0) but not near-neutral (pH 7.5) growth medium. Acidic pH promotes PBP6b production, specific activity, and stability (55).

**(ii) Intracellular pathogens carry genes that encode pH specialist cell wall enzymes.** pH-sensitive cell wall enzymes have also been identified in Salmonella enterica serotype Typhimurium and Mycobacterium tuberculosis (62–65). Although not environmental generalists like E. coli, these pathogens experience pH stress in host intracellular compartments, wherein they persist and replicate.

M. tuberculosis and *S.* Typhimurium both carry genes that encode acid-responsive dl-endopeptidases required for persistence in the phagosome (62, 65). M. tuberculosis genes encode two semiredundant dl-endopeptidases, RipA and RipB. While production of either enzyme is sufficient to support normal growth of M. tuberculosis in rich medium at neutral pH, RipA becomes conditionally essential for growth in acidic medium and in chronic infections (64, 65). RipA abundance is regulated through MarP, a periplasmic serine protease required to maintain intracellular pH homeostasis (66). MarP-mediated processing of RipA is required for its hydrolytic activity. Consistent with RipA’s increased importance in acidic environments, MarP-RipA interaction is strengthened in low-pH medium (65). Similarly, *S.* Typhimurium produces a dl-endopeptidase, EcgA, specifically required for growth when cells are cultured in epithelial cells or in low-pH minimal medium. EgcA is required for the pathogen to efficiently colonize the livers and spleens of infected mice, suggesting this enzyme facilitates adaptation to its intracellular lifestyle (62).

Castanheira and colleagues identified an unusual acid-regulated cell wall enzyme in *S.* Typhimurium (63). Unlike laboratory E. coli, which produces a single PBP3 enzyme essential for cytokinesis (28), *S.* Typhimurium produces two: a conventional PBP3 enzyme produced from the *dcw* cluster with other division proteins and a second copy at a distal locus with 63% identity to the aforementioned enzyme (designated PBP3_SAL_). PBP3_SAL_ is specifically produced and active in acidic environments, including during replication in the phagosome. Strikingly, continuous culturing in acidic medium permits inactivation of PBP3, indicating that PBP3_SAL_ can substitute for PBP3’s essential function in cytokinesis (63). PBP3_SAL_ has a reduced affinity for β-lactam antibiotics compared to PBP3 and may contribute to relapsing infections following antibiotic treatment (63, 67).

Redundancy among “essential” components of the cell division and elongation machinery may not be unique to *Salmonella*. Pseudomonas aeruginosa also carries a gene that encodes a second PBP3 homolog, PBP3x, which is upregulated in stationary phase. Similar to PBP3_SAL_, PBP3x does not bind β-lactam antibiotics as well as the conventional copy (63, 68). Likewise, Listeria monocytogenes genes encode multiple copies of the SEDS glycosyltransferases, RodA (3 homologs) and FtsW (2 homologs). While not transcribed under normal laboratory conditions, *rodA3* and *ftsW2* are expressed when cells are exposed to cell envelope stress, such as when grown in the presence of subinhibitory concentrations of β-lactam antibiotics (69). In both P. aeruginosa or L. monocytogenes, however, it remains unclear whether the aforementioned redundant homologs can functionally substitute for the preferred cognate class B PBP/SEDS enzymes or whether they instead perform specialized roles in cell wall biogenesis.

**(iii) Zinc specialist endopeptidases in Vibrio cholerae and Acinetobacter baumannii.** Beyond pH, metal limitation restricts the growth of bacterial pathogens. Several classes of cell wall autolysins require a zinc cofactor for catalysis, yet sequestration by the host restricts the availability of this metal *in vivo*. One conserved adaptation to this adverse condition appears to be the production of zinc-regulated autolysins. Two complementary studies recently identified upregulated genes encoding cell wall autolysins in V. cholerae and A. baumannii in zinc-limiting environments (70, 71).

V. cholerae genes encode three endopeptidases, ShyA to ShyC, which have overlapping roles in cell growth and morphogenesis. Only *shyA* and *shyC* are expressed in rich growth medium, and inactivation of both is lethal under this condition (72). Their combined absence, however, is tolerated in zinc restrictive medium due to Zur-dependent expression of *shyB* (70). Like ShyA and ShyC, ShyB likely requires a zinc cofactor for catalysis. ShyB activity, however, is more resistant to zinc chelators *in vitro* than the other two enzymes. This property may confer a fitness benefit in zinc restrictive environments. Accordingly, the ShyB homolog in A. baumannii, ZrlA, is required for colonization and dissemination in a murine model of pneumonia (71). Interestingly, although a Δ*zrlA* mutant has increased cell envelope permeability, it is defective for zinc uptake, suggesting that this enzyme may also have a specialized role in ensuring zinc transporters are inserted in the cell envelope. Consistent with this model, *zrlA* is located adjacent to and may be cotranscribed with *znuA*, which encodes an essential component of the zinc uptake system. This genomic context is conserved for homologs in many gammaproteobacteria, including for the E. coli enzyme MepM (70), suggesting a conserved role in metal homeostasis.

### Modulatory factors maintain integrity of cell wall synthesis machines.

While the majority of cell wall enzymes are believed to function autonomously or through transient interactions with a small number of regulators, some enzymes are active only in the context of large cell wall synthesis machines, comprised of many protein-protein interactions. In bacteria, multicomponent cell wall synthesis complexes include the divisome, the Rod system, and cortical PG apparatus. While the Rod system and cortical PG apparatus are restricted to rod-shaped and spore-forming bacteria, respectively, the divisome is conserved in nearly all bacteria with a cell wall. The molecular players may vary between species, but in general, the divisome consists of cell wall synthesis enzymes and regulators, which are scaffolded by treadmilling filaments of the tubulin homolog FtsZ at midcell (25, 26). Divisome proteins can be classified based on essential function(s), with individual factors performing one or more roles in (i) localization of other cytokinetic machinery components to midcell, (ii) stabilization of the divisome complex, and (iii) synthesis of new septal PG. In the previous section, we examined the contribution of enzymatic redundancy to robust PG synthesis. Here, we review a second adaptation to preserve divisome function and integrity across growth conditions: the production of stabilization and modulatory factors that are preferentially required for division during growth in particular environmental conditions. For a comprehensive overview of divisome structure and function more generally, we recommend two excellent reviews (73, 74).

**(i) Condition-dependent modulatory proteins in E. coli.** Significant genetic data from many groups indicate that division proteins FtsEX, ZipA, FtsK, FtsN, FtsP, and DedD all share partially overlapping roles in stabilizing, and potentially activating, septal PG synthesis in E. coli (75–80). While many of these proteins promote efficient division across culture conditions, several are strictly required for viability only during growth in particular environmental conditions. FtsEX and FtsP, for example, are conditionally essential in media with low osmotic strength and at high temperature (75, 81, 82). Among other roles, FtsEX and FtsP share an overlapping function in divisome stabilization and thus cannot be simultaneously inactivated under any osmotic condition (75, 82). Remarkably, these factors share no structural relatedness: the FtsEX complex—a predicted ABC transporter—is anchored in the inner membrane through FtsX’s transmembrane domain, while FtsP is a soluble periplasmic protein belonging to the multicopper oxidase family (75, 81, 83). Further complicating the picture, FtsEX and FtsP share partial functional overlap with the “essential” division protein FtsN. Overexpression of *ftsN* bypasses the requirement for FtsP and FtsEX in low-osmotic-strength medium (75, 82), as well as circumvents the essential function of FtsK in cytokinesis under standard culture conditions (76). Like FtsP and FtsEX, the strict requirement for wild-type concentrations of FtsN and FtsK is conditional, dependent on the pH of the growth medium. Cells tolerate depletion—and even complete loss—of FtsK in acidic medium, a condition which corresponds with enrichment of FtsN at the cytokinetic ring. In accordance with the critical function for FtsN at low pH, it cannot be depleted in acidic medium (80), even in genetic backgrounds which permit extensive depletion at neutral pH (84–86).

**(ii) PerM stabilizes the mycobacterial divisome during chronic infection.** Not surprisingly, conditionally essential divisome proteins also exist outside E. coli. Notably, work from the Ehrt group recently identified a new actinobacterial integral membrane division protein, PerM, which is preferentially required for cytokinesis in persistent M. tuberculosis infections (87). Mutants defective for PerM replicate more slowly, are longer, and exhibit cell separation defects *in vivo*, specifically during the chronic phase of a murine infection model (88). In culture, the Δ*perM* mutant is also sensitive to acidic pH, magnesium concentration, and iron limitation—physiologically relevant stresses the bacterium encounters when persisting within activated macrophages in chronic infection (88, 89). PerM functions by stabilizing the essential division protein FtsB, and phenotypes associated with PerM loss are alleviated through *ftsB* overexpression. Surprisingly, FtsB abundance in wild-type cells did not differ across stress conditions (88), suggesting that FtsB activity or stoichiometry in the divisome may be condition dependent.

**(iii) Hints of condition-dependent modulatory proteins outside the divisome.** It remains unclear whether condition-specific factors stabilize other specialized cell wall synthesis machines, like the Rod system. Compared to the divisome, the Rod system has fewer higher-order interactions and is under less strict spatiotemporal regulation. These qualities may alleviate the need for stabilization factors altogether. On the other hand, there is some evidence to suggest conditional essentiality of Rod system components in Staphylococcus aureus and Streptococcus pneumoniae. Although S. aureus and S. pneumoniae are spherical and lack the actin homolog MreB, both organisms carry genes that encode nonenzymatic Rod system components MreC and MreD, factors recently implicated in the activation of peripheral cell wall synthesis enzymes in E. coli (18). MreC and MreD are essential in virulent, but not laboratory, isolates of S. pneumoniae (90). Likewise, while MreC and MreD are dispensable for PG synthesis and cell shape of S. aureus in the laboratory setting (91), mutants defective for their production are specifically attenuated in a chronic murine abscess model (92). Similar to the mycobacterial protein PerM, S. aureus *ΔmreC* and *ΔmreD* mutants did not have a growth defect in acute infection environments relative to the parental strain (92), suggesting that these proteins are required for maximal reproductive fitness only when exposed to a chronic infection-related stressor.

## RESPONSES TO ACUTE CELL WALL DAMAGE

Acute stress that threatens PG integrity is an equally—if not more—formidable challenge to cell wall integrity than growth in nonoptimal conditions. Defects in PG integrity are typically lethal, resulting in cell lysis and death within minutes. Both intrinsic and extrinsic sources can damage PG by introducing gaps into the sacculus. Intrinsic sources of damage include the insertion and removal of envelope-spanning complexes (e.g., lipopolysaccharide [LPS] transport machinery) or defects in coupling between cell wall synthase and activity. Extrinsic sources of damage are more diverse and include chemical agents (e.g., β-lactam or glycopeptide antibiotics), host antimicrobial defenses (e.g., lysozyme), exogenous transfer machinery (e.g., type six secretion or conjugation machinery), predators (e.g., phage or predatory bacteria), and mechanical stress (e.g., shear force).

Regardless of origin, acute threats to PG integrity are mitigated through a specific damage response. The first and most immediate step is lesion repair to prevent osmotic rupture. Subsequent to lesion repair, additional mechanisms may be initiated to fortify the cell wall (e.g., PG modifications) or alter the cell’s developmental program (e.g., dormancy or L-form switching) to protect the cell against recurrent damage (93, 94). Here, we review recent developments in our understanding of the immediate response with a specific focus on the role of E. coli enzyme PBP1b in this process. The latter, longer timescale responses to acute damage have been discussed in depth by others, and we direct the reader to these reviews for additional information (93, 94).

### Evidence that PBP1b mediates PG repair in E. coli.

Several pieces of evidence implicate a role for the bifunctional class A PBPs, and specially PBP1b, in E. coli cell wall quality control. (i) Class A PBP dynamics suggest a unique role in cell wall synthesis compared to other PBPs (11, 95). (ii) PBP1b activity is elevated in response to cell envelope stress (95, 96). (iii) PBP1b is required for survival and recovery from cell wall damage (95, 97, 98).

Single molecule studies provided the first experimental evidence the class A PBPs function outside the cytoskeletal machinery and may play a role in cell wall quality control. The PG synthesis enzymes associated with the Rod complex or the divisome move directionally around the cell body (22–27, 99) and insert “hoops” of PG oriented roughly perpendicular to the long axis of the cell (100, 101). In contrast, class A PBPs in E. coli and B. subtilis do not exhibit ballistic motion. Instead, individual molecules transition between diffusive and immobile states, which represent inactive and active enzymes, respectively (11, 102). Moreover, unlike the Rod system and divisome, active class A PBPs insert disordered PG material throughout the cell body (11, 95, 100, 101). Based on these data, it was proposed the class A PBPs fill in the gaps within the PG scaffold synthesized by the Rod system and divisome (2, 11). Analogous to building a house, the Rod system and divisome assemble the framework that supports its structure and provides its shape, whereas the class A PBPs fill in the gaps to insulate and seal the structure off from the environment.

**FIG 2:**
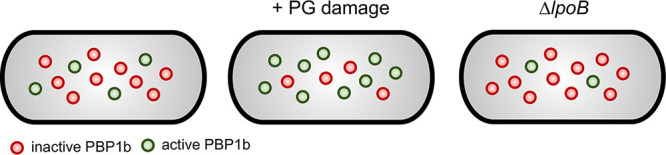
Model of PBP1b activity in response to peptidoglycan damage. The fraction of PBP1b molecules actively engaged in peptidoglycan metabolism varies in response to environmental damage through a mechanism that is dependent on its outer membrane lipoprotein activator LpoB.

Additional data indicate that PBP1b responds to changes in PG integrity in E. coli. Just as more material is required to fill a more open framework, greater PBP1b activity is required in response to an increase in number or size of gaps within the PG matrix (Fig. 2). Reductions in PG density through PG precursor depletion increase the immobile (active) fraction of PBP1b (95). Likewise, overproduction of the dd-endopeptidase MepS—whose activity increases the rate of cell surface expansion (38)—stimulates PBP1b activity when other PG synthases are inactivated (96). Further consistent with a role in PG repair, PBP1b is required for efficient recovery from damaging agents. Mutants defective for PBP1b exhibited a reduced ability to recover from precursor depletion compared to the parental strain (95). Moreover, loss of PBP1b or its cofactor LpoB render cells hypersensitive to β-lactam treatment (13, 103), mechanical stress (98), and outer membrane damage (33).

### Localization determinants of PBP1b.

How does PBP1b recognize gaps in a macromolecule considerably larger in size? One possibility is that class A PBP enzymes exist in multicomponent protein complexes composed of cell wall synthases and autolysins (60, 104). Direct interactions between two classes of enzymes would ensure that their activities are coupled and spatially restricted to regions of cell wall growth. Consistent with this model, the outer membrane lipoprotein NlpI scaffolds several endopeptidases and PBP1a (105). It is unknown, however, whether direct interactions with autolysins are required for synthase activity *in vivo*, at least in the case of PBP1b (96). Moreover, it is not clear how complex formation would assist in PBP1b recognition and repair of gaps in the PG matrix unrelated to activity such as during PG precursor depletion or upon exogenous cell wall damage (95).

Alternatively, PBP1b may recognize sites of cell wall damage directly, as has been shown for the E. coli Rod system transpeptidase PBP2 (106), or through interaction with its cognate outer membrane lipoprotein activator LpoB. Structural analysis of LpoB suggests a potential mechanism for “sensing” and recruiting PBP1b to cell wall pores. LpoB possesses a long, intrinsically disordered N-terminal linker connected to the C-terminal globular domain, which interacts with and activates PBP1b (107–110). Conformational heterogeneity of the linker may permit extension into PG gaps and temporarily trap the globular domain, thereby enriching PBP1b activity at porous regions in the PG (2, 12). Consistent with this model, LpoB is required for efficient PBP1b immobilization and survival during recovery from cell wall stress (95) (Fig. 2).

### Do other class A penicillin binding proteins promote peptidoglycan quality control?

While these data support a role for PBP1b in closing gaps in the PG, it remains unclear whether PBP1a also plays a role in E. coli cell wall quality control. Unlike PBP1b, loss of PBP1a does not sensitize cells to β-lactam antibiotics or mechanical damage in otherwise wild-type cells (13, 98, 103). Furthermore, excess PBP1a production cannot maintain normal mechanical integrity of the cell wall in the absence of PBP1b (95, 98). Altogether, these data suggest while PBP1a and PBP1b have overlapping roles in exponential growth, PBP1b may have a unique function in cell wall quality control. It is tempting to speculate that this difference may result in part from structural differences between their cognate outer membrane lipoprotein activators. Unlike LpoB, LpoA possesses lower interdomain flexibility and exists solely in an extended conformation (111).

Whether class A PBPs play a conserved role in cell wall quality control outside E. coli also remains unknown. While class A PBPs are broadly conserved in nearly all bacteria with a cell wall, PBP1b homologs have been lost in some lineages such as the beta- and alphaproteobacteria (12), suggesting the existence of alternative quality control systems in these organisms. LpoB conservation is even more limited: LpoB homologs are found only in a subset of gammaproteobacteria (12, 112). Interestingly, unique class A PBP cofactors have evolved independently in some Gram-negative and actinobacteria (112, 113), some of which also possess intrinsically disordered linkers (114). Mechanisms of cell wall quality control are equally, if not more, mysterious in Gram-positive bacteria. Class A PBP activity is not strictly essential in several Gram-positive bacteria (115, 116), and in B. subtilis, these enzymes play roles in width control not shared by their E. coli counterparts (95, 101).

### Additional factors implicated in E. coli cell wall quality control.

Several additional enzymes have been implicated in E. coli PG repair, including the ld-transpeptidase LdtD and the carboxypeptidases PBP5 and PBP6a (33). Morè et al. determined that genes encoding all three enzymes and PBP1b are upregulated in response to outer membrane stress induced by depletion of an essential component of the LPS transport machinery (33, 117). Consistent with a role in maintaining PG integrity, mutants defective for any of these enzymes are unable to survive LPS depletion, eventually forming membrane bulges before lysing altogether. Only the glycosyltransferase activity of PBP1b is required to protect cells from lysis (33). These data suggest a model in which PBP5 or PBP6b trims the peptide stems on the nascent glycan strands synthesized by PBP1b, thereby providing a substrate for transpeptidation and the formation of 3-3 cross-links by LdtD. The authors propose that these enzymes function together as a PG repair complex, required to fill gaps in the sacculus caused by loss of the envelope spanning LPS transport machinery. It remains unclear whether PBP1b, LdtD, PBP5, and PBP6a play a conserved role in cell wall repair or whether their activity is specific to damage incurred during outer membrane stress. With the exception of PBP5, loss of either PBP6a or LdtD does not sensitize cells to β-lactam antibiotics or mechanical damage (98, 103).

## CONCLUDING REMARKS

Like other essential cellular processes, PG metabolism must remain robust in response to exogenous and endogenous stressors to preserve cell viability. Plasticity with the active repertoire of PG synthases, autolysins, and regulators ensures continuity of PG metabolism even in unfavorable growth conditions. PG quality control enzymes repair lesions in the cell’s protective armor in response to acute damaging agents. At the cell surface, these processes represent attractive therapeutic targets. Small molecules that target PBP1b or disarm other components of PG quality control machinery may potentiate the activity of other cell wall active antibiotics. Likewise, the development of condition-specific therapeutics may increase efficient clearance of bacterial infections in particular host niches, while sparing members of the normal flora. Future studies on robustness in PG metabolism in other bacterial pathogens and nonmodel organisms with disparate shapes, sizes, and growth and developmental patterns promise to illuminate additional interesting biology and therapeutic targets. We anticipate that the mechanisms bacteria use to fortify and repair their cell wall are as diverse as the environments they inhabit.

## References

[1] Vollmer W, Seligman SJ 2010 Architecture of peptidoglycan: more data and more models. Trends Microbiol 18:59–66. doi:10.1016/j.tim.2009.12.004. 20060721

[2] Typas A, Banzhaf M, Gross CA, Vollmer W 2011 From the regulation of peptidoglycan synthesis to bacterial growth and morphology. Nat Rev Microbiol 10:123–136. doi:10.1038/nrmicro2677. 22203377 PMC5433867

[3] Barreteau H, Kovač A, Boniface A, Sova M, Gobec S, Blanot D 2008 Cytoplasmic steps of peptidoglycan biosynthesis. FEMS Microbiol Rev 32:168–207. doi:10.1111/j.1574-6976.2008.00104.x. 18266853

[4] Sham L-T, Butler EK, Lebar MD, Kahne D, Bernhardt TG, Ruiz N 2014 MurJ is the flippase of lipid-linked precursors for peptidoglycan biogenesis. Science 345:220–222. doi:10.1126/science.1254522. 25013077 PMC4163187

[5] Meeske AJ, Sham L-T, Kimsey H, Koo B-M, Gross CA, Bernhardt TG, Rudner DZ 2015 MurJ and a novel lipid II flippase are required for cell wall biogenesis in Bacillus subtilis. Proc Natl Acad Sci U S A 112:6437–6442. doi:10.1073/pnas.1504967112. 25918422 PMC4443310

[6] Egan AJF, Errington J, Vollmer W 2020 Regulation of peptidoglycan synthesis and remodelling. Nat Rev Microbiol 18:446–460. doi:10.1038/s41579-020-0366-3. 32424210

[7] Gupta R, Lavollay M, Mainardi J-L, Arthur M, Bishai WR, Lamichhane G 2010 The Mycobacterium tuberculosis protein LdtMt2 is a nonclassical transpeptidase required for virulence and resistance to amoxicillin. Nat Med 16:466–469. doi:10.1038/nm.2120. 20305661 PMC2851841

[8] Mainardi J-L, Hugonnet J-E, Rusconi F, Fourgeaud M, Dubost L, Moumi AN, Delfosse V, Mayer C, Gutmann L, Rice LB, Arthur M 2007 Unexpected inhibition of peptidoglycan LD-transpeptidase from Enterococcus faecium by the β-lactam imipenem. J Biol Chem 282:30414–30422. doi:10.1074/jbc.M704286200. 17646161

[9] Suginaka H, Blumberg PM, Strominger JL 1972 Multiple penicillin-binding components in Bacillus subtilis, Bacillus cereus, Staphylococcus aureus, and Escherichia coli. J Biol Chem 247:5279–5288. 4626716

[10] Yousif SY, Broome-Smith JK, Spratt BG 1985 Lysis of Escherichia coli by beta-lactam antibiotics: deletion analysis of the role of penicillin-binding proteins 1A and 1B. J Gen Microbiol 131:2839–2845. doi:10.1099/00221287-131-10-2839. 3906031

[11] Cho H, Wivagg CN, Kapoor M, Barry Z, Rohs PDA, Suh H, Marto JA, Garner EC, Bernhardt TG 2016 Bacterial cell wall biogenesis is mediated by SEDS and PBP polymerase families functioning semi-autonomously. Nat Microbiol 1:16172. doi:10.1038/nmicrobiol.2016.172. 27643381 PMC5030067

[12] Typas A, Banzhaf M, van Saparoea B, Verheul J, Biboy J, Nichols RJ, Zietek M, Beilharz K, Kannenberg K, von Rechenberg M, Breukink E, den Blaauwen T, Gross CA, Vollmer W 2010 Regulation of peptidoglycan synthesis by outer-membrane proteins. Cell 143:1097–1109. doi:10.1016/j.cell.2010.11.038. 21183073 PMC3060616

[13] Paradis-Bleau C, Markovski M, Uehara T, Lupoli TJ, Walker S, Kahne DE, Bernhardt TG 2010 Lipoprotein cofactors located in the outer membrane activate bacterial cell wall polymerases. Cell 143:1110–1120. doi:10.1016/j.cell.2010.11.037. 21183074 PMC3085243

[14] Schiffer G, Höltje J-V 1999 Cloning and characterization of PBP 1C, a third member of the multimodular class A penicillin-binding proteins of Escherichia coli. J Biol Chem 274:32031–32039. doi:10.1074/jbc.274.45.32031. 10542235

[15] Budd A, Blandin S, Levashina EA, Gibson TJ 2004 Bacterial alpha2-macroglobulins: colonization factors acquired by horizontal gene transfer from the metazoan genome? Genome Biol 5:R38. doi:10.1186/gb-2004-5-6-r38. 15186489 PMC463071

[16] Doan N, Gettins PGW 2008 Alpha-macroglobulins are present in some gram-negative bacteria: characterization of the alpha2-macroglobulin from Escherichia coli. J Biol Chem 283:28747–28756. doi:10.1074/jbc.M803127200. 18697741 PMC2568910

[17] Meeske AJ, Riley EP, Robins WP, Uehara T, Mekalanos JJ, Kahne D, Walker S, Kruse AC, Bernhardt TG, Rudner DZ 2016 SEDS proteins are a widespread family of bacterial cell wall polymerases. Nature 537:634–638. doi:10.1038/nature19331. 27525505 PMC5161649

[18] Rohs PDA, Buss J, Sim SI, Squyres GR, Srisuknimit V, Smith M, Cho H, Sjodt M, Kruse AC, Garner EC, Walker S, Kahne DE, Bernhardt TG 2018 A central role for PBP2 in the activation of peptidoglycan polymerization by the bacterial cell elongation machinery. PLoS Genet 14:e1007726. doi:10.1371/journal.pgen.1007726. 30335755 PMC6207328

[19] Taguchi A, Welsh MA, Marmont LS, Lee W, Sjodt M, Kruse AC, Kahne D, Bernhardt TG, Walker S 2019 FtsW is a peptidoglycan polymerase that is functional only in complex with its cognate penicillin-binding protein. Nat Microbiol 4:587–594. doi:10.1038/s41564-018-0345-x. 30692671 PMC6430707

[20] Doi M, Wachi M, Ishino F, Tomioka S, Ito M, Sakagami Y, Suzuki A, Matsuhashi M 1988 Determinations of the DNA sequence of the mreB gene and of the gene products of the mre region that function in formation of the rod shape of Escherichia coli cells. J Bacteriol 170:4619–4624. doi:10.1128/JB.170.10.4619-4624.1988. 3049542 PMC211501

[21] Tamaki S, Matsuzawa H, Matsuhashi M 1980 Cluster of mrdA and mrdB genes responsible for the rod shape and mecillinam sensitivity of Escherichia coli. J Bacteriol 141:52–57. doi:10.1128/JB.141.1.52-57.1980. 6243629 PMC293528

[22] van Teeffelen S, Wang S, Furchtgott L, Huang KC, Wingreen NS, Shaevitz JW, Gitai Z 2011 The bacterial actin MreB rotates, and rotation depends on cell-wall assembly. Proc Natl Acad Sci U S A 108:15822–15827. doi:10.1073/pnas.1108999108. 21903929 PMC3179079

[23] Garner EC, Bernard R, Wang W, Zhuang X, Rudner DZ, Mitchison T 2011 Coupled, circumferential motions of the cell wall synthesis machinery and MreB filaments in B. subtilis. Science 333:222–225. doi:10.1126/science.1203285. 21636745 PMC3235694

[24] Domínguez-Escobar J, Chastanet A, Crevenna AH, Fromion V, Wedlich-Söldner R, Carballido-López R 2011 Processive movement of MreB-associated cell wall biosynthetic complexes in bacteria. Science 333:225–228. doi:10.1126/science.1203466. 21636744

[25] Bisson-Filho AW, Hsu Y-P, Squyres GR, Kuru E, Wu F, Jukes C, Sun Y, Dekker C, Holden S, VanNieuwenhze MS, Brun YV, Garner EC 2017 Treadmilling by FtsZ filaments drives peptidoglycan synthesis and bacterial cell division. Science 355:739–743. doi:10.1126/science.aak9973. 28209898 PMC5485650

[26] Yang X, Lyu Z, Miguel A, McQuillen R, Huang KC, Xiao J 2017 GTPase activity–coupled treadmilling of the bacterial tubulin FtsZ organizes septal cell wall synthesis. Science 355:744–747. doi:10.1126/science.aak9995. 28209899 PMC5851775

[27] Yang X, McQuillen R, Lyu Z, Phillips-Mason P, Cruz ADL, McCausland JW, Liang H, DeMeester KE, Grimes CL, de Boer P, Xiao J 2019 FtsW exhibits distinct processive movements driven by either septal cell wall synthesis or FtsZ treadmilling in E. coli. bioRxiv 10.1101/850073.

[28] Spratt BG 1975 Distinct penicillin binding proteins involved in the division, elongation, and shape of Escherichia coli K12. Proc Natl Acad Sci U S A 72:2999–3003. doi:10.1073/pnas.72.8.2999. 1103132 PMC432906

[29] Boyle DS, Khattar MM, Addinall SG, Lutkenhaus J, Donachie WD 1997 ftsW is an essential cell‐division gene in Escherichia coli. Mol Microbiol 24:1263–1273. doi:10.1046/j.1365-2958.1997.4091773.x. 9218774

[30] Khattar MM, Begg KJ, Donachie WD 1994 Identification of FtsW and characterization of a new ftsW division mutant of Escherichia coli. J Bacteriol 176:7140–7147. doi:10.1128/jb.176.23.7140-7147.1994. 7961485 PMC197100

[31] Wang L, Khattar MK, Donachie WD, Lutkenhaus J 1998 FtsI and FtsW are localized to the septum in Escherichia coli. J Bacteriol 180:2810–2816. doi:10.1128/JB.180.11.2810-2816.1998. 9603865 PMC107242

[32] Magnet S, Bellais S, Dubost L, Fourgeaud M, Mainardi J-L, Petit-Frère S, Marie A, Mengin-Lecreulx D, Arthur M, Gutmann L 2007 Identification of the l,d-transpeptidases responsible for attachment of the Braun lipoprotein to Escherichia coli peptidoglycan. J Bacteriol 189:3927–3931. doi:10.1128/JB.00084-07. 17369299 PMC1913343

[33] Morè N, Martorana AM, Biboy J, Otten C, Winkle M, Serrano CKG, Silva AM, Atkinson L, Yau H, Breukink E, den Blaauwen T, Vollmer W, Polissi A 2019 Peptidoglycan remodeling enables Escherichia coli to survive severe outer membrane assembly defect. mBio 10:e02729-18. [CrossRef] doi:10.1128/mBio.02729-18. 30723128 PMC6428754

[34] Glauner B, Höltje JV, Schwarz U 1988 The composition of the murein of Escherichia coli. J Biol Chem 263:10088–10095. 3292521

[35] Hara H, Suzuki H 1984 A novel glycan polymerase that synthesizes uncross-linked peptidoglycan in Escherichia coli. FEBS Lett 168:155–160. doi:10.1016/0014-5793(84)80226-4. 6368264

[36] Do T, Page JE, Walker S 2020 Uncovering the activities, biological roles, and regulation of bacterial cell wall hydrolases and tailoring enzymes. J Biol Chem 295:3347–3361. doi:10.1074/jbc.REV119.010155. 31974163 PMC7062177

[37] Singh SK, SaiSree L, Amrutha RN, Reddy M 2012 Three redundant murein endopeptidases catalyse an essential cleavage step in peptidoglycan synthesis of Escherichia coli K12. Mol Microbiol 86:1036–1051. doi:10.1111/mmi.12058. 23062283

[38] Oldewurtel ER, Kitahara Y, Cordier B, Özbaykal G, van Teeffelen S 2019 Bacteria control cell volume by coupling cell-surface expansion to dry-mass growth. bioRxiv 10.1101/769786.

[39] Heidrich C, Templin MF, Ursinus A, Merdanovic M, Berger J, Schwarz H, Pedro MAD, Höltje J-V 2001 Involvement of N-acetylmuramyl-L-alanine amidases in cell separation and antibiotic-induced autolysis of Escherichia coli: cell separation and antibiotic-induced autolysis of E. coli. Mol Microbiol 41:167–178. doi:10.1046/j.1365-2958.2001.02499.x. 11454209

[40] Priyadarshini R, de Pedro MA, Young KD 2007 Role of peptidoglycan amidases in the development and morphology of the division septum in Escherichia coli. J Bacteriol 189:5334–5347. doi:10.1128/JB.00415-07. 17483214 PMC1951850

[41] Heidrich C, Ursinus A, Berger J, Schwarz H, Holtje J-V 2002 Effects of multiple deletions of murein hydrolases on viability, septum cleavage, and sensitivity to large toxic molecules in Escherichia coli. J Bacteriol 184:6093–6099. doi:10.1128/jb.184.22.6093-6099.2002. 12399477 PMC151956

[42] Priyadarshini R, Popham DL, Young KD 2006 Daughter cell separation by penicillin-binding proteins and peptidoglycan amidases in Escherichia coli. J Bacteriol 188:5345–5355. doi:10.1128/JB.00476-06. 16855223 PMC1540038

[43] Weaver AI, Jiménez‐Ruiz V, Tallavajhala SR, Ransegnola BP, Wong KQ, Dörr T 2019 Lytic transglycosylases RlpA and MltC assist in Vibrio cholerae daughter cell separation. Mol Microbiol 112:1100–1115. doi:10.1111/mmi.14349. 31286580 PMC6800776

[44] Nelson DE, Young KD 2001 Contributions of PBP 5 and dd-carboxypeptidase penicillin binding proteins to maintenance of cell shape in Escherichia coli. J Bacteriol 183:3055–3064. doi:10.1128/JB.183.10.3055-3064.2001. 11325933 PMC95205

[45] Nelson DE, Young KD 2000 Penicillin binding protein 5 affects cell diameter, contour, and morphology of Escherichia coli. J Bacteriol 182:1714–1721. doi:10.1128/jb.182.6.1714-1721.2000. 10692378 PMC94470

[46] Yunck R, Cho H, Bernhardt TG 2016 Identification of MltG as a potential terminase for peptidoglycan polymerization in bacteria. Mol Microbiol 99:700–718. doi:10.1111/mmi.13258. 26507882 PMC4752859

[47] Kraft AR, Prabhu J, Ursinus A, Höltje JV 1999 Interference with murein turnover has no effect on growth but reduces beta-lactamase induction in Escherichia coli. J Bacteriol 181:7192–7198. doi:10.1128/JB.181.23.7192-7198.1999. 10572120 PMC103679

[48] Park JT, Uehara T 2008 How bacteria consume their own exoskeletons (turnover and recycling of cell wall peptidoglycan). Microbiol Mol Biol Rev 72:211–227. doi:10.1128/MMBR.00027-07. 18535144 PMC2415748

[49] Yamamoto S, Tsukamoto T, Terai A, Kurazono H, Takeda Y, Yoshida O 1997 Genetic evidence supporting the fecal-perineal-urethral hypothesis in cystitis caused by Escherichia coli. J Urol 157:1127–1129. doi:10.1016/S0022-5347(01)65154-1. 9072556

[50] Wood JM 2011 Bacterial osmoregulation: a paradigm for the study of cellular homeostasis. Annu Rev Microbiol 65:215–238. doi:10.1146/annurev-micro-090110-102815. 21663439

[51] Slonczewski JL, Fujisawa M, Dopson M, Krulwich TA 2009 Cytoplasmic pH measurement and homeostasis in bacteria and archaea. Adv Microb Physiol 55:1–79, 317. doi:10.1016/S0065-2911(09)05501-5. 19573695

[52] Wilks JC, Slonczewski JL 2007 pH of the cytoplasm and periplasm of Escherichia coli: rapid measurement by green fluorescent protein fluorimetry. J Bacteriol 189:5601–5607. doi:10.1128/JB.00615-07. 17545292 PMC1951819

[53] Chakraborty S, Winardhi RS, Morgan LK, Yan J, Kenney LJ 2017 Non-canonical activation of OmpR drives acid and osmotic stress responses in single bacterial cells. Nat Commun 8:1587. doi:10.1038/s41467-017-02030-0. 29138484 PMC5686162

[54] Rosenbusch JP 1990 Structural and functional properties of porin channels in E. coli outer membranes. Experientia 46:167–173. 1689255

[55] Peters K, Kannan S, Rao VA, Biboy J, Vollmer D, Erickson SW, Lewis RJ, Young KD, Vollmer W 2016 The redundancy of peptidoglycan carboxypeptidases ensures robust cell shape maintenance in Escherichia coli. mBio 7:e00819-16. doi:10.1128/mBio.00819-16. 27329754 PMC4916385

[56] Dai X, Zhu M 2018 High osmolarity modulates bacterial cell size through reducing initiation volume in Escherichia coli. mSphere 3:e00430-18. doi:10.1128/mSphere.00430-18. 30355666 PMC6200984

[57] Mueller EA, Egan AJ, Breukink E, Vollmer W, Levin PA 2019 Plasticity of Escherichia coli cell wall metabolism promotes fitness and antibiotic resistance across environmental conditions. Elife 8:e40754. doi:10.7554/eLife.40754. 30963998 PMC6456298

[58] Suzuki H, Nishimura Y, Hirota Y 1978 On the process of cellular division in Escherichia coli: a series of mutants of E. coli altered in the penicillin-binding proteins. Proc Natl Acad Sci U S A 75:664–668. doi:10.1073/pnas.75.2.664. 345275 PMC411316

[59] Reed P, Atilano ML, Alves R, Hoiczyk E, Sher X, Reichmann NT, Pereira PM, Roemer T, Filipe SR, Pereira-Leal JB, Ligoxygakis P, Pinho MG 2015 Staphylococcus aureus survives with a minimal peptidoglycan synthesis machine but sacrifices virulence and antibiotic resistance. PLoS Pathog 11:e1004891. doi:10.1371/journal.ppat.1004891. 25951442 PMC4423922

[60] Pazos M, Peters K, Vollmer W 2017 Robust peptidoglycan growth by dynamic and variable multi-protein complexes. Curr Opin Microbiol 36:55–61. doi:10.1016/j.mib.2017.01.006. 28214390

[61] Egan AJF, Vollmer W 2015 The stoichiometric divisome: a hypothesis. Front Microbiol 6:455. doi:10.3389/fmicb.2015.00455. 26029191 PMC4428075

[62] Rico-Pérez G, Pezza A, Pucciarelli MG, de Pedro MA, Soncini FC, Portillo FG 2016 A novel peptidoglycan D,L-endopeptidase induced by Salmonella inside eukaryotic cells contributes to virulence. Mol Microbiol 99:546–556. doi:10.1111/mmi.13248. 26462856

[63] Castanheira S, Cestero JJ, Rico-Pérez G, García P, Cava F, Ayala JA, Pucciarelli MG, Portillo FG 2017 A specialized peptidoglycan synthase promotes Salmonella cell division inside host cells. mBio 8:e01685-17. doi:10.1128/mBio.01685-17. 29259085 PMC5736910

[64] Healy C, Gouzy A, Ehrt S 2020 Peptidoglycan hydrolases RipA and Ami1 are critical for replication and persistence of Mycobacterium tuberculosis in the host. mBio 11:e03315-19. [CrossRef] doi:10.1128/mBio.03315-19. 32127458 PMC7064781

[65] Botella H, Vaubourgeix J, Lee MH, Song N, Xu W, Makinoshima H, Glickman MS, Ehrt S 2017 Mycobacterium tuberculosis protease MarP activates a peptidoglycan hydrolase during acid stress. EMBO J 36:536–548. doi:10.15252/embj.201695028. 28057704 PMC5437814

[66] Vandal OH, Roberts JA, Odaira T, Schnappinger D, Nathan CF, Ehrt S 2009 Acid-susceptible mutants of Mycobacterium tuberculosis share hypersusceptibility to cell wall and oxidative stress and to the host environment. J Bacteriol 191:625–631. doi:10.1128/JB.00932-08. 19011036 PMC2620805

[67] Castanheira S, López-Escarpa D, Pucciarelli MG, Cestero JJ, Baquero F, Portillo FG 2020 An alternative penicillin-binding protein involved in Salmonella relapses following ceftriaxone therapy. Ebiomedicine 55:102771. doi:10.1016/j.ebiom.2020.102771. 32344200 PMC7186495

[68] Liao X, Hancock RE 1997 Identification of a penicillin-binding protein 3 homolog, PBP3x, in Pseudomonas aeruginosa: gene cloning and growth phase-dependent expression. J Bacteriol 179:1490–1496. doi:10.1128/jb.179.5.1490-1496.1997. 9045804 PMC178857

[69] Rismondo J, Halbedel S, Gründling A 2019 Cell shape and antibiotic resistance are maintained by the activity of multiple FtsW and RodA enzymes in Listeria monocytogenes. mBio 10:e01448-19. doi:10.1128/mBio.01448-19. 31387909 PMC6686043

[70] Murphy SG, Alvarez L, Adams MC, Liu S, Chappie JS, Cava F, Dörr T 2019 Endopeptidase regulation as a novel function of the Zur-dependent zinc starvation response. mBio 10:e02620-18. [CrossRef] doi:10.1128/mBio.02620-18. 30782657 PMC6381278

[71] Lonergan ZR, Nairn BL, Wang J, Hsu Y-P, Hesse LE, Beavers WN, Chazin WJ, Trinidad JC, VanNieuwenhze MS, Giedroc DP, Skaar EP 2019 An Acinetobacter baumannii, zinc-regulated peptidase maintains cell wall integrity during immune-mediated nutrient sequestration. Cell Rep 26:2009–2018.e6. doi:10.1016/j.celrep.2019.01.089. 30784584 PMC6441547

[72] Dörr T, Cava F, Lam H, Davis BM, Waldor MK 2013 Substrate specificity of an elongation-specific peptidoglycan endopeptidase and its implications for cell wall architecture and growth of Vibrio cholerae. Mol Microbiol 89:949–962. doi:10.1111/mmi.12323. 23834664 PMC3769093

[73] Haeusser DP, Margolin W 2016 Splitsville: structural and functional insights into the dynamic bacterial Z ring. Nat Rev Microbiol 14:305–319. doi:10.1038/nrmicro.2016.26. 27040757 PMC5290750

[74] Du S, Lutkenhaus J 2017 Assembly and activation of the Escherichia coli divisome. Mol Microbiol 105:177–187. doi:10.1111/mmi.13696. 28419603 PMC5517055

[75] Samaluru H, SaiSree L, Reddy M 2007 Role of SufI (FtsP) in cell division of Escherichia coli: evidence for Its involvement in stabilizing the assembly of the divisome. J Bacteriol 189:8044–8052. doi:10.1128/JB.00773-07. 17766410 PMC2168700

[76] Goehring NW, Robichon C, Beckwith J 2007 Role for the nonessential N terminus of FtsN in divisome assembly. J Bacteriol 189:646–649. doi:10.1128/JB.00992-06. 17071748 PMC1797402

[77] Du S, Pichoff S, Lutkenhaus J 2016 FtsEX acts on FtsA to regulate divisome assembly and activity. Proc Natl Acad Sci U S A 113:E5052–E5061. doi:10.1073/pnas.1606656113. 27503875 PMC5003251

[78] Pichoff S, Du S, Lutkenhaus J 2018 Disruption of divisome assembly rescued by FtsN-FtsA interaction in Escherichia coli. Proc Natl Acad Sci U S A 115:E6855–E6862. doi:10.1073/pnas.1806450115. 29967164 PMC6055203

[79] Liu B, Hale CA, Persons L, Phillips-Mason PJ, de Boer PAJ 2019 Roles of the DedD protein in Escherichia coli cell constriction. J Bacteriol 201:e00698-18. [CrossRef] doi:10.1128/JB.00698-18. 30692172 PMC6436348

[80] Mueller EA, Westfall CS, Levin PA 2020 pH-dependent activation of cytokinesis modulates Escherichia coli cell size. PLoS Genet 16:e1008685. doi:10.1371/journal.pgen.1008685. 32203516 PMC7117782

[81] Schmidt KL, Peterson ND, Kustusch RJ, Wissel MC, Graham B, Phillips GJ, Weiss DS 2004 A predicted ABC transporter, FtsEX, is needed for cell division in Escherichia coli. J Bacteriol 186:785–793. doi:10.1128/jb.186.3.785-793.2004. 14729705 PMC321481

[82] Reddy M 2007 Role of FtsEX in cell division of Escherichia coli: viability of ftsEX mutants is dependent on functional SufI or high osmotic strength. J Bacteriol 189:98–108. doi:10.1128/JB.01347-06. 17071757 PMC1797223

[83] Arends SJR, Kustusch RJ, Weiss DS 2009 ATP-binding site lesions in FtsE impair cell division. J Bacteriol 191:3772–3784. doi:10.1128/JB.00179-09. 19376877 PMC2698383

[84] Geissler B, Shiomi D, Margolin W 2007 The ftsA* gain-of-function allele of Escherichia coli and its effects on the stability and dynamics of the Z ring. Microbiology (Reading) 153:814–825. doi:10.1099/mic.0.2006/001834-0. 17322202 PMC4757590

[85] Liu B, Persons L, Lee L, Boer PAJ 2015 Roles for both FtsA and the FtsBLQ subcomplex in FtsN‐stimulated cell constriction in Escherichia coli. Mol Microbiol 95:945–970. doi:10.1111/mmi.12906. 25496160 PMC4428282

[86] Tsang M, Bernhardt TG 2015 A role for the FtsQLB complex in cytokinetic ring activation revealed by an ftsL allele that accelerates division. Mol Microbiol 95:925–944. doi:10.1111/mmi.12905. 25496050 PMC4414402

[87] Goodsmith N, Guo XV, Vandal OH, Vaubourgeix J, Wang R, Botella H, Song S, Bhatt K, Liba A, Salgame P, Schnappinger D, Ehrt S 2015 Disruption of an M. tuberculosis membrane protein causes a magnesium-dependent cell division defect and failure to persist in mice. PLoS Pathog 11:e1004645. doi:10.1371/journal.ppat.1004645. 25658098 PMC4450064

[88] Wang R, Kreutzfeldt K, Botella H, Vaubourgeix J, Schnappinger D, Ehrt S 2019 Persistent Mycobacterium tuberculosis infection in mice requires PerM for successful cell division. Elife 8:e49570. doi:10.7554/eLife.49570. 31751212 PMC6872210

[89] Ehrt S, Schnappinger D 2009 Mycobacterial survival strategies in the phagosome: defence against host stresses. Cell Microbiol 11:1170–1178. doi:10.1111/j.1462-5822.2009.01335.x. 19438516 PMC3170014

[90] Land AD, Winkler ME 2011 The requirement for pneumococcal MreC and MreD is relieved by inactivation of the gene encoding PBP1a. J Bacteriol 193:4166–4179. doi:10.1128/JB.05245-11. 21685290 PMC3147673

[91] Tavares AC, Fernandes PB, Carballido-López R, Pinho MG 2015 MreC and MreD proteins are not required for growth of Staphylococcus aureus. PLoS One 10:e0140523. doi:10.1371/journal.pone.0140523. 26470021 PMC4607420

[92] Valentino MD, Foulston L, Sadaka A, Kos VN, Villet RA, Maria JS, Lazinski DW, Camilli A, Walker S, Hooper DC, Gilmore MS 2014 Genes contributing to Staphylococcus aureus fitness in abscess- and infection-related ecologies. mBio 5:e01729-14. doi:10.1128/mBio.01729-14. 25182329 PMC4173792

[93] Yadav AK, Espaillat A, Cava F 2018 Bacterial strategies to preserve cell wall integrity against environmental threats. Front Microbiol 9:2064. doi:10.3389/fmicb.2018.02064. 30233540 PMC6127315

[94] Claessen D, Errington J 2019 Cell wall deficiency as a coping strategy for stress. Trends Microbiol 27:1025–1033. doi:10.1016/j.tim.2019.07.008. 31420127

[95] Vigouroux A, Cordier B, Aristov A, Alvarez L, Özbaykal G, Chaze T, Oldewurtel ER, Matondo M, Cava F, Bikard D, van Teeffelen S 2020 Class-A penicillin binding proteins do not contribute to cell shape but repair cell-wall defects. Elife 9:e51998. doi:10.7554/eLife.51998. 31904338 PMC7002073

[96] Lai GC, Cho H, Bernhardt TG 2017 The mecillinam resistome reveals a role for peptidoglycan endopeptidases in stimulating cell wall synthesis in Escherichia coli. PLoS Genet 13:e1006934. doi:10.1371/journal.pgen.1006934. 28749938 PMC5549755

[97] Ranjit DK, Jorgenson MA, Young KD 2017 PBP1B glycosyltransferase and transpeptidase activities play different essential roles during the de novo regeneration of rod morphology in Escherichia coli. J Bacteriol 199:e00612-16. doi:10.1128/JB.00612-16. 28096447 PMC5350282

[98] Auer GK, Lee TK, Rajendram M, Cesar S, Miguel A, Huang KC, Weibel DB 2016 Mechanical genomics identifies diverse modulators of bacterial cell stiffness. Cell Syst 2:402–411. doi:10.1016/j.cels.2016.05.006. 27321372 PMC4967499

[99] Perez AJ, Cesbron Y, Shaw SL, Villicana JB, Tsui H-CT, Boersma MJ, Ye ZA, Tovpeko Y, Dekker C, Holden S, Winkler ME 2019 Movement dynamics of divisome proteins and PBP2x:FtsW in cells of Streptococcus pneumoniae. Proc Natl Acad Sci U S A 116:3211–3220. doi:10.1073/pnas.1816018116. 30718427 PMC6386697

[100] Turner RD, Mesnage S, Hobbs JK, Foster SJ 2018 Molecular imaging of glycan chains couples cell-wall polysaccharide architecture to bacterial cell morphology. Nat Commun 9:1263. doi:10.1038/s41467-018-03551-y. 29593214 PMC5871751

[101] Dion MF, Kapoor M, Sun Y, Wilson S, Ryan J, Vigouroux A, van Teeffelen S, Oldenbourg R, Garner EC 2019 Bacillus subtilis cell diameter is determined by the opposing actions of two distinct cell wall synthetic systems. Nat Microbiol 4:1294–1305. doi:10.1038/s41564-019-0439-0. 31086310 PMC6656618

[102] Lee TK, Meng K, Shi H, Huang KC 2016 Single-molecule imaging reveals modulation of cell wall synthesis dynamics in live bacterial cells. Nat Commun 7:13170. doi:10.1038/ncomms13170. 27774981 PMC5078992

[103] Nichols RJ, Sen S, Choo YJ, Beltrao P, Zietek M, Chaba R, Lee S, Kazmierczak KM, Lee KJ, Wong A, Shales M, Lovett S, Winkler ME, Krogan NJ, Typas A, Gross CA 2011 Phenotypic landscape of a bacterial cell. Cell 144:143–156. doi:10.1016/j.cell.2010.11.052. 21185072 PMC3060659

[104] Höltje J-V 1993 “Three for one”—a simple growth mechanism that guarantees a precise copy of the thin, rod-shaped murein sacculus of Escherichia coli, p 419–426. *In* de Pedro MA, Höltje JV, Löffelhardt W (ed), Bacterial growth and lysis—metabolism and structure of the bacterial sacculus. Plenum, New York, NY.

[105] Banzhaf M, Yau HC, Verheul J, Lodge A, Kritikos G, Mateus A, Cordier B, Hov AK, Stein F, Wartel M, Pazos M, Solovyova AS, Breukink E, van Teeffelen S, Savitski MM, den Blaauwen T, Typas A, Vollmer W 2020 Outer membrane lipoprotein NlpI scaffolds peptidoglycan hydrolases within multi-enzyme complexes in Escherichia coli. EMBO J 39:e102246. doi:10.15252/embj.2019102246. 32009249 PMC7049810

[106] Özbaykal G, Wollrab E, Simon F, Vigouroux A, Cordier B, Aristov A, Chaze T, Matondo M, van Teeffelen S 2020 The transpeptidase PBP2 governs initial localization and activity of the major cell-wall synthesis machinery in E. coli. Elife 9:e50629. doi:10.7554/eLife.50629. 32077853 PMC7089770

[107] Egan AJF, Jean NL, Koumoutsi A, Bougault CM, Biboy J, Sassine J, Solovyova AS, Breukink E, Typas A, Vollmer W, Simorre J-P 2014 Outer-membrane lipoprotein LpoB spans the periplasm to stimulate the peptidoglycan synthase PBP1B. Proc Natl Acad Sci U S A 111:8197–8202. doi:10.1073/pnas.1400376111. 24821816 PMC4050580

[108] King DT, Lameignere E, Strynadka NCJ 2014 Structural insights into the lipoprotein outer membrane regulator of penicillin-binding protein 1B. J Biol Chem 289:19245–19253. doi:10.1074/jbc.M114.565879. 24808177 PMC4081958

[109] Markovski M, Bohrhunter JL, Lupoli TJ, Uehara T, Walker S, Kahne DE, Bernhardt TG 2016 Cofactor bypass variants reveal a conformational control mechanism governing cell wall polymerase activity. Proc Natl Acad Sci U S A 113:4788–4793. doi:10.1073/pnas.1524538113. 27071112 PMC4855605

[110] Egan AJF, Maya-Martinez R, Ayala I, Bougault CM, Banzhaf M, Breukink E, Vollmer W, Simorre J-P 2018 Induced conformational changes activate the peptidoglycan synthase PBP1B. Mol Microbiol 110:335–356. doi:10.1111/mmi.14082. 30044025 PMC6220978

[111] Jean NL, Bougault CM, Lodge A, Derouaux A, Callens G, Egan AJF, Ayala I, Lewis RJ, Vollmer W, Simorre J-P 2014 Elongated structure of the outer-membrane activator of peptidoglycan synthesis LpoA: implications for PBP1A stimulation. Structure 22:1047–1054. doi:10.1016/j.str.2014.04.017. 24954617 PMC4111904

[112] Greene NG, Fumeaux C, Bernhardt TG 2018 Conserved mechanism of cell-wall synthase regulation revealed by the identification of a new PBP activator in Pseudomonas aeruginosa. Proc Natl Acad Sci U S A 115:3150–3155. doi:10.1073/pnas.1717925115. 29507210 PMC5866570

[113] Sher JW, Lim HC, Bernhardt TG 2020 Global phenotypic profiling identifies a conserved actinobacterial cofactor for a bifunctional PBP-type cell wall synthase. Elife 9:e54761. doi:10.7554/eLife.54761. 32167475 PMC7205459

[114] Caveney NA, Egan AJF, Ayala I, Laguri C, Robb CS, Breukink E, Vollmer W, Strynadka NCJ, Simorre J-P 2020 Structure of the peptidoglycan synthase activator LpoP in Pseudomonas aeruginosa. Structure 28:643–650.e5. doi:10.1016/j.str.2020.03.012. 32320673 PMC7267771

[115] McPherson DC, Popham DL 2003 Peptidoglycan synthesis in the absence of class A penicillin-binding proteins in Bacillus subtilis. J Bacteriol 185:1423–1431. doi:10.1128/jb.185.4.1423-1431.2003. 12562814 PMC142859

[116] Rice LB, Carias LL, Rudin S, Hutton R, Marshall S, Hassan M, Josseaume N, Dubost L, Marie A, Arthur M 2009 Role of class A penicillin-binding proteins in the expression of beta-lactam resistance in Enterococcus faecium. J Bacteriol 191:3649–3656. doi:10.1128/JB.01834-08. 19304851 PMC2681891

[117] Martorana AM, Motta S, Silvestre DD, Falchi F, Dehò G, Mauri P, Sperandeo P, Polissi A 2014 Dissecting Escherichia coli outer membrane biogenesis using differential proteomics. PLoS One 9:e100941. doi:10.1371/journal.pone.0100941. 24967819 PMC4072712

[118] Paradis-Bleau C, Kritikos G, Orlova K, Typas A, Bernhardt TG 2014 A genome-wide screen for bacterial envelope biogenesis mutants identifies a novel factor involved in cell wall precursor metabolism. PLoS Genet 10:e1004056. doi:10.1371/journal.pgen.1004056. 24391520 PMC3879167

[119] Ursinus A, Höltje JV 1994 Purification and properties of a membrane-bound lytic transglycosylase from Escherichia coli. J Bacteriol 176:338–343. doi:10.1128/jb.176.2.338-343.1994. 8288527 PMC205055

[120] Lommatzsch J, Templin MF, Kraft AR, Vollmer W, Höltje JV 1997 Outer membrane localization of murein hydrolases: MltA, a third lipoprotein lytic transglycosylase in Escherichia coli. J Bacteriol 179:5465–5470. doi:10.1128/jb.179.17.5465-5470.1997. 9287002 PMC179418

[121] Dörr T, Möll A, Chao MC, Cava F, Lam H, Davis BM, Waldor MK 2014 Differential requirement for PBP1a and PBP1b in in vivo and in vitro fitness of Vibrio cholerae. Infect Immun 82:2115–2124. doi:10.1128/IAI.00012-14. 24614657 PMC3993429

[122] Möll A, Dörr T, Alvarez L, Chao MC, Davis BM, Cava F, Waldor MK 2014 Cell separation in Vibrio cholerae is mediated by a single amidase whose action is modulated by two nonredundant activators. J Bacteriol 196:3937–3948. doi:10.1128/JB.02094-14. 25182499 PMC4248829

[123] Hocking J, Priyadarshini R, Takacs CN, Costa T, Dye NA, Shapiro L, Vollmer W, Jacobs-Wagner C 2012 Osmolality-dependent relocation of penicillin-binding protein PBP2 to the division site in Caulobacter crescentus. J Bacteriol 194:3116–3127. doi:10.1128/JB.00260-12. 22505677 PMC3370875

[124] Fujimoto M, Goto R, Hirota R, Ito M, Haneda T, Okada N, Miki T 2018 Tat-exported peptidoglycan amidase-dependent cell division contributes to Salmonella Typhimurium fitness in the inflamed gut. PLoS Pathog 14:e1007391. doi:10.1371/journal.ppat.1007391. 30379938 PMC6231687

[125] Patru M-M, Pavelka MS 2010 A role for the class A penicillin-binding protein PonA2 in the survival of Mycobacterium smegmatis under conditions of nonreplication. J Bacteriol 192:3043–3054. doi:10.1128/JB.00025-10. 20400545 PMC2901687

[126] Sureka K, Hossain T, Mukherjee P, Chatterjee P, Datta P, Kundu M, Basu J 2010 Novel role of phosphorylation-dependent interaction between FtsZ and FipA in mycobacterial cell division. PLoS One 5:e8590. doi:10.1371/journal.pone.0008590. 20066037 PMC2797604

[127] Foulquier E, Pompeo F, Bernadac A, Espinosa L, Galinier A 2011 The YvcK protein is required for morphogenesis via localization of PBP1 under gluconeogenic growth conditions in Bacillus subtilis. Mol Microbiol 80:309–318. doi:10.1111/j.1365-2958.2011.07587.x. 21320184

[128] Sassine J, Xu M, Sidiq KR, Emmins R, Errington J, Daniel RA 2017 Functional redundancy of division specific penicillin‐binding proteins in Bacillus subtilis. Mol Microbiol 106:304–318. doi:10.1111/mmi.13765. 28792086 PMC5656894

